# Association of Opioid Prescription with Major Adverse Cardiovascular Events: Nationwide Cohort Study

**DOI:** 10.3390/jcm14041205

**Published:** 2025-02-12

**Authors:** Tak-Kyu Oh, Hyoung-Won Cho, In-Ae Song

**Affiliations:** 1 Department of Anesthesiology and Pain Medicine, Seoul National University Bundang Hospital, Seongnam 13620, Republic of Korea; airohtak@hotmail.com; 2 Department of Anesthesiology and Pain Medicine, College of Medicine, Seoul National University, Seoul 03080, Republic of Korea; 3 Department of Cardiology, Seoul National University Hospital, Seoul 03080, Republic of Korea

**Keywords:** analgesics, opioid, cardiovascular diseases, mortality

## Abstract

**Background**: This study aimed to investigate the association between opioid use and the incidence of major adverse cardiovascular events (MACEs). **Methods**: This study included adult patients who had received oral or transdermal opioids in 2016. The control group comprised individuals who did not receive opioids in 2016 and was selected using a 1:1 stratified random sampling procedure. A MACE was defined as the occurrence of acute myocardial infarction, stroke, heart failure, or cardiovascular mortality. The primary endpoints were new MACEs and cardiovascular mortality, as evaluated from 1 January 2017 to 31 December 2021. **Results**: The study included 4,179,130 participants, of whom 1,882,945 (45.1%) were opioid users. After propensity score matching, 1,811,732 individuals (905,866 in each group) were included. Cox regression analysis revealed that the opioid user group had a 24% higher incidence of MACEs than the non-user group (hazard ratio [HR]: 1.24; 95% confidence interval [CI]: 1.23, 1.24; *p* < 0.001). Additionally, the opioid user group showed a 30% higher risk of cardiovascular mortality than the non-user group (HR: 1.30; 95% CI: 1.26, 1.35; *p* < 0.001). **Conclusions**: Opioid use was associated with an increased incidence of MACE and higher risk of cardiovascular mortality.

## 1. Introduction

Opioids are widely used as analgesics and pain relievers [1]. However, their use is associated with an increased risk of dependency and addiction [2], resulting in a global opioid crisis [3]. The opioid crisis has now become one of the most serious public health concerns, and according to a recent study, the risk of opioid misuse and dependency is greatest in Australia, Canada, France, Germany, the United Kingdom, and the United States [4]. Moreover, the European Society of Anaesthesiology and Intensive Care emphasizes the significance of rigorous preoperative evaluation of patients receiving opioids, considering their potential impact on perioperative outcomes [5].

Major adverse cardiovascular events (MACEs) are usually defined as acute myocardial infarction (AMI), stroke, heart failure (HF), and cardiovascular mortality [6,7]. They are a major public health concern because the total prevalence of cardiovascular diseases nearly doubled from 271 million in 1990 to 523 million in 2019, and the number of deaths due to these diseases consistently increased from 12.1 million in 1990 to 18.6 million in 2019 [8]. The relationship between opioid use and the risk of adverse cardiovascular events has been reported previously [9]. Synthetic opioids can cause pro-arrhythmias, and opioid agonism and antagonism/withdrawal have been associated with the development of severe cardiovascular events [9]. Therefore, opioid administration may be a risk factor for MACE development. However, there is insufficient evidence on this issue. Hence, this study aimed to investigate whether oral or transdermal opioid use is associated with the incidence of MACEs in South Korea. We hypothesized that opioid use would increase the incidence of MACEs.

## 2. Materials and Methods

### 2.1. Study Design and Ethical Statement

The recommendations for Strengthening the Reporting of Observational Studies in Epidemiology were followed in this population-based cohort study [10]. The study protocol was excluded from discussion by the institutional review board because of the use of publicly available data. The study’s institutional review board approval number is X-2307-840-903. The study protocol was approved by the National Health Insurance Agency (NHIS) (approval number NHIS-2023-1-115), and data access authorization was obtained. All procedures were carried out in accordance with the ethical standards established by national and institutional human experimentation committees, as well as the principles of the Helsinki Declaration of 1975, which was amended in 2008. The institutional review board waived the requirement for informed consent because of the use of retroactively collected anonymized data.

### 2.2. Data Source

South Korea’s NHIS is the sole provider of public health insurance. It gathers and stores detailed information on drug prescriptions, treatments, and disease diagnoses. The information is arranged and classified using International Classification of Diseases, 10th Revision (ICD-10) codes. The NHIS is a government-run healthcare system in South Korea that requires foreign residents who have stayed in the country for more than 6 months to register. Furthermore, the NHIS database contains information on the socioeconomic factors and death rates of all individuals [11].

### 2.3. Study Population

**Figure 1:**
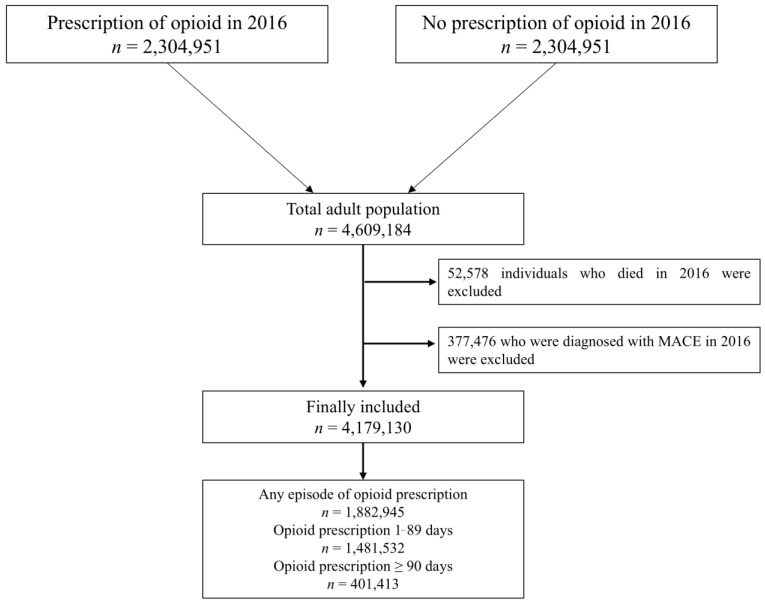
Flow chart depicting the selection process for study participants.

Initially, data for all adults (age ≥ 20 years) who obtained opioid prescriptions from medical facilities between 1 January 2016 and 31 December 2016, were requested. Data for each individual were collected for only 1 day of opioid medication. Consequently, this study included 2,304,592 individuals who received opioid prescriptions in 2016. Subsequently, a 1:1 stratified random sampling technique, taking age and sex into account, was used to request data for 2,304,592 adult non-users who did not obtain any opioid prescriptions between 1 January 2016 and 31 December 2016. Thus, the study included 4,609,184 adults. After 52,578 people who died in 2016 and 377,476 who were diagnosed with MACEs in 2016 were eliminated, 4,179,130 individuals were included in the analysis. In 2016, 1,882,945 individuals (45.1%) were prescribed opioids. The numbers of individuals who were administered opioids for 1–89 and 90 days were 1,481,532 (32.1%) and 401,413 (9.6%), respectively. Figure 1 shows the participant selection process used in this study.

### 2.4. Opioid Users

In South Korea, all drug prescriptions by physicians are recorded and managed by the National Centralized Prescription Management System of the NHIS. Therefore, there are no cases of missing prescriptions for opioids. We defined opioid users as those who were prescribed oral or transdermal opioids, excluding intravenous opioids. In South Korea, intravenous opioid analgesics are typically administered only at medical institutions. Consequently, individuals requiring long-term opioid use are generally prescribed oral or transdermal forms for home administration. The oral form of opioids also included a combination of opioids and paracetamol, given the explosive increase in tramadol/acetaminophen combination products in South Korea [12]. According to the classification criteria for short- and long-term opioid prescription periods, participants were divided into three groups: non-users, 1- to 89-day opioid users, and ≥90-day opioid users [13]. To assess opioid prescriptions (lasting more than three months), data on opioid prescriptions from October 2015 to March 2017 were analyzed. Because the study period was just one year, this technique was chosen to reduce the misclassification of persons who received long-term prescriptions as short-term users. For example, patients who received opioid prescriptions between November 2016 and February 2017 may have been incorrectly classified as short-term users. The selection procedure for the research population is illustrated in Figure 1.

### 2.5. Study Endpoint (MACEs and Cardiovascular Mortality in 2017–2021)

Based on the previous literature [6,7], a MACE was defined as the occurrence of AMI, HF, or stroke. AMI was defined as hospitalization with a primary or secondary diagnosis of ICD-10 codes I21-23. After hospitalization, HF was defined using the discharge diagnosis ICD-10 codes of I11.0, I13.0, I13.2, I25.5, I42, I50, and O90.3. Stroke was defined using the discharge diagnosis ICD-10 codes of I60-64 among hospitalized patients who had undergone brain imaging procedures, such as computed tomography and magnetic resonance imaging [14]. Cardiovascular mortality was defined using the ICD-10 codes I00-I99 for the main cause of death. In South Korea, the main cause of death needs to be registered by physicians, and these data were provided by Statistics Korea. Both MACEs and cardiovascular mortality were evaluated from 1 January 2017 to 31 December 2021.

### 2.6. Covariate Analysis

The demographic data included age and sex. Furthermore, data on the socioeconomic positions, household income levels, and residences of the study population were obtained. Household income levels were divided into six groups: medical aid program, unknown group, and four quartile ratio groups. Generally, individuals in the unknown group whose household income is not captured by the NHIS are more likely to be military personnel or undocumented immigrants. The government classifies individuals who are unable to pay insurance premiums because of poverty into a medical aid program group.

The capital and other metropolitan cities were designated as urban, whereas the rest of the country was classified as rural. Because all disabilities must be reported in the NHIS database to obtain various benefits from South Korea’s social welfare programs, information about underlying disabilities was gathered. All disabilities need to be officially determined by a professional doctor based on difficulties in performing daily activities. Underlying disabilities were classified according to severity as mild, moderate, and severe. To determine patients’ comorbid status, the Elixhauser Comorbidity Index was obtained for 29 underlying conditions [15]. Since other analgesics, such as paracetamol, nonsteroidal anti-inflammatory drugs, gabapentin, and pregabalin, were also prescribed, prescription data for these analgesics were collected.

### 2.7. Statistical Methodology

The clinicopathological characteristics of the study population are presented as mean values with standard deviation (SD) for continuous variables and as numbers with percentages for categorical variables. Although the non-user group without opioid prescriptions in 2016 was extracted by a medical records technician at the NHIS center through stratified random sampling, taking age and sex into account, the opioid user and non-user groups had many differences. Therefore, we attempted to balance the groups through propensity score (PS) matching for other characteristics besides sex and age [16], including household income level, residence, underlying disability, and 29 underlying comorbid conditions. In particular, PS matching was executed without replacement and with a caliper width of 0.25, employing the nearest neighbor method in a 1:1 ratio. The absolute value of the standardized mean difference (ASD) was used to assess the balance between the two groups after PS matching. An ASD below 0.2 implied that PS matching was suitable. Although PS matching was performed, we compared the characteristics of the two groups using the Chi-square test and *t*-test.

In the PS-matched cohort, we performed Cox regression analyses for the incidences of overall MACEs, specific MACEs (AMI, HF, and stroke), and cardiovascular mortality. Moreover, hazard plots of the incidence of cardiovascular mortality in the PS-matched groups were created using Kaplan–Meir Estimation, with a log-rank test measuring statistical significance.

Additionally, we performed a sensitivity analysis using multivariable Cox regression modeling to examine whether the results obtained in the PS-matched cohort were similar to those of the entire cohort. All the covariates were included in the multivariable-adjusted model. The occurrence of MACEs or cardiovascular mortality was set as an event in the models, and the duration from 1 January 2017, to the date of the MACE diagnosis or cardiovascular death was considered the duration in the time-to-event analysis. Additional multivariable Cox regression models were employed to predict the occurrence of MACEs or cardiovascular mortality, with opioid users divided into two separate subgroups: those who had used opioids for 1–89 days and those who had used opioids for 90 days. The effects of the duration of opioid prescription on patient outcomes were also investigated. All results are reported as hazard ratios (HRs) with 95% confidence intervals (CIs), and log–log plots were used to confirm that the key assumptions of the Cox proportional hazard models were met. At a variance inflation factor of 2, no multicollinearity between the variables was explored using the multivariable model. SPSS for Windows (version 250; IBM Corp., Armonk, NY, USA) was used for all statistical analyses, and statistical significance was set at *p* < 0.05.

## 3. Results

### 3.1. Study Population

The clinicopathological characteristics of the study population before and after PS matching are shown in Table 1. After PS matching, the ASD was less than 0.2 for all variables, indicating that PS matching was successful. Although a *t*-test or Chi-square test shows a significant difference in the PS-matched cohort, this could be attributed to the large sample size (over 1.8 million). However, the ASD is typically used to assess the balance between two groups after PS matching [17].

### 3.2. Incidence of MACE and Cardiovascular Mortality in the PS-Matched Cohort

**Figure 2:**
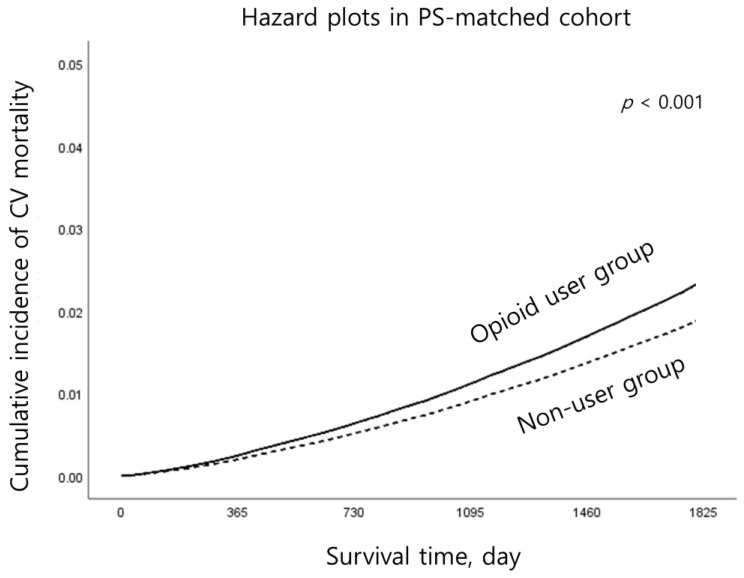
Hazard plots of the cumulative incidence of cardiovascular mortality in the two groups. PS—propensity score; CV—cardiovascular.

Table 2 shows the results of MACE incidence and cardiovascular mortality before and after PS matching. Follow-up data were collected for both MACEs and cardiovascular mortality for 5 years from 1 January 2017 to 31 December 2021. In the PS-matched cohort, the total incidence rates of MACEs in 2017–2021 in the opioid user and non-user groups were 20.0% (181,448/905,866) and 16.4% (148,565/905,866), respectively. In Cox regression, the opioid user group showed a 24% higher incidence of MACEs than the non-user group (HR: 1.24, 95% CI: 1.23, 1.24; *p* < 0.001). Specifically, the opioid user group showed a 32% (HR: 1.32, 95% CI: 1.30, 1.34; *p* < 0.001), 25% (HR: 1.25, 95% CI: 1.24, 1.27; *p* < 0.001), and 23% (HR: 1.23, 95% CI: 1.21, 1.24; *p* < 0.001) higher incidence of AMI, HF, and stroke than the non-user group, respectively. In addition, the opioid user group showed a 30% higher risk of cardiovascular mortality than the non-user group (HR: 1.30, 95% CI: 1.26, 1.35; *p* < 0.001). Figure 2 shows the hazard plots of the cumulative incidence of cardiovascular mortality in the two groups, and the opioid user group showed higher cumulative incidence than the non-user group (*p* < 0.001 by log-rank test).

### 3.3. Sensitivity Analysis in the Entire Cohort

Table 3 shows the results of the multivariable Cox regression model for MACEs or cardiovascular mortality in 2017–2021. The opioid user group showed a 12% higher incidence of MACEs than the non-user group (HR: 1.12, 95% CI: 1.10, 1.12; *p* < 0.001; model 1). Both long- and short-term opioid users had 18% (HR: 1.18, 95% CI: 1.17, 1.19; *p* < 0.001; model 2) and 10% (HR: 1.10, 95% CI: 1.09, 1.10; *p* < 0.001; model 2) higher incidences of MACEs, respectively, than the non-user group. The opioid user group showed a 16% higher incidence of AMI than the non-user group (HR: 1.16, 95% CI: 1.12, 1.15; *p* < 0.001; model 3). Both long- and short-term opioid users showed a 10% (HR: 1.23, 95% CI: 1.21, 1.25; *p* < 0.001; model 4) and 14% (HR: 1.14, 95% CI: 1.12, 1.15; *p* < 0.001; model 4) higher incidence of MACEs, respectively, than the non-user group. The opioid user group showed a 12% higher incidence of HF than the non-user group (HR: 1.12, 95% CI: 1.11, 1.13; *p* < 0.001; model 5). Both long- and short-term opioid users had 18% (HR: 1.18, 95% CI: 1.17, 1.19; *p* < 0.001; model 6) and 9% (HR: 1.09, 95% CI: 1.08, 1.10; *p* < 0.001; model 6) higher incidences of HF, respectively, than the non-user group. The opioid user group showed a 9% higher incidence of stroke than the non-user group (HR: 1.09, 95% CI: 1.08, 1.10; *p* < 0.001; model 7). Both long- and short-term opioid users showed a 13% (HR: 1.13, 95% CI: 1.12, 1.15; *p* < 0.001; model 8) and 7% (HR: 1.07, 95% CI: 1.05, 1.08; *p* < 0.001; model 8) higher incidence of stroke, respectively, than the non-user group.

The opioid user group showed a 19% higher incidence of cardiovascular mortality than the non-user group (HR: 1.19, 95% CI: 1.15, 1.22; *p* < 0.001; model 3). Both long- and short-term opioid users showed 11% (HR: 1.11, 95% CI: 1.07, 1.14; *p* < 0.001; model 3) and 32% (HR: 1.18, 95% CI: 1.27, 1.37; *p* < 0.001; model 4) higher incidences of cardiovascular mortality, respectively, than the non-user group. All other covariates with HRs and 95% CIs in multivariable models 1 and 3 are presented in Supplementary Tables S1 and S2.

## 4. Discussion

Compared with non-users, opioid users were associated with higher incidence rates of both MACEs and cardiovascular mortality in our population-based cohort analysis. Furthermore, this connection was stronger among long-term opioid users than among short-term users. The data suggest that opioid prescriptions may increase the incidence of MACEs in adults, emphasizing the importance of weighing the benefits and dangers of such prescriptions. Our data show that preventing opioid abuse or misuse is critical for lowering the incidence of MACEs and related death.

In our study, the high prevalence of short-term opioid use among the adult population in South Korea is noteworthy. Patients in South Korea have relatively high access to healthcare facilities and can readily receive opioid prescriptions, given the broad coverage by the National Health Insurance Agency [18]. Therefore, the present study’s results should be interpreted carefully, and comparison with findings from other countries is warranted.

The cardiovascular effects of chronic opioid use are still being investigated [9]. This phenomenon has been explained using several theories. First, opioid use is known to cause androgen deficiency as an endocrine effect of opioids [19,20], and sex hormones may exert a considerable influence on the cardiovascular system, such as in the form of hyperlipidemia [21]. Second, opioids can increase platelet aggregation, which can increase the risk of thrombosis through increased plasminogen activator inhibitor-1 and plasma fibrinogen [22]. Third, oxidative stress is a significant factor in the development of atherosclerosis. Morphine and heroin have been shown to reduce total antioxidant capacity. Elevated oxidative stress may be implicated in atherosclerosis among opium users [23].

Setoguchi et al. reported that current opioid use and the cumulative use of 11 or more prescriptions were associated with a slightly elevated risk of myocardial infarction (MI) compared with non-use, with the risk being higher among morphine, meperidine, and polytherapy users [24]. This phenomenon has been explained using several theories. In this context, according to Solomon et al., compared with nonselective NSAID use, opioid use was associated with a 2.25-fold increased risk of MI in older adults [25]. Moreover, chronic opioid therapy (cumulative use for 180 days) was related to an elevated incidence rate ratio of 2.66 (95% CI: 2.30, 3.08) for MI compared with that in the general population, according to Carman et al. [26]. We used AMI to define MACEs and showed that the incidence of AMI could increase in both short- and long-term opioid users.

Khodneva et al. reported that opioid prescriptions were associated with increased MACEs, such as coronary heart disease and cardiovascular mortality among female individuals [27]. Moreover, Singleton et al. reported that long-term opioid use and opioid use disorders were associated with the risk of cardiovascular conditions such as MI, congestive HF, cardiac arrhythmia (including cardiac arrest), and stroke [28]. Chronic opioid use may be linked to higher levels of low-density lipoproteins and triglycerides, which may lead to coronary artery disease [29]. A previous study in rats revealed that chronic opioid use was associated with alterations in the serum concentrations of triglycerides and total high-density lipoprotein and low-density lipoprotein cholesterol [30]. Hyperlipidemia is a known risk factor for atherosclerosis, leading to MI or stroke [31,32].

Opioid use also increased the risk of mortality in patients with HF in a meta-analysis of previously published research [33], and a more recent review reported that current opioid use was associated with a high risk of in-hospital mortality in patients with acute HF [34], However, no study has definitively focused on the relationship between opioid use and the incidence of HF. Our results suggest that opioid use could be a potential risk factor for the development of HF during long-term follow-up. There have been mixed results regarding the relationship between opioid use and stroke risk. Lee et al. discovered an association between morphine use for cancer pain and hemorrhagic stroke but not ischemic stroke [35]. From 2008 to 2015, the incidence rate of stroke among hospitalized patients increased by 20.3% with the addition of opioid misuse, according to Omran et al. [36]. However, Khodneva et al. found no overall association between prescription opioid use for chronic non-cancerous pain and the development of stroke [27]. Our study showed that both short- and long-term opioid use could be related to an increased incidence of stroke in a large sample of participants from South Korea. Further studies are required to confirm the relationship between opioid prescriptions and the incidence of HF or stroke.

This study has several limitations. First, opioid doses were not considered. Second, owing to a lack of information in the NHIS database, many relevant factors, such as body mass index, smoking history, and alcohol intake, were not included as covariates. Third, because our study used data from the South Korean national registration database, generalizability to other countries may be limited. Fourth, unmeasured or residual variables may have influenced the findings. Fifth, some opioid users in 2016 may have stopped using opioids during the study period (2017–2021), whereas non-users in 2016 may have begun using opioids during this period, which may have influenced our findings. Sixth, the results of this study may have been influenced by the health insurance system in South Korea, hindering generalizability to other countries. Lastly, we did not consider the specific type of opioid and route of opioid administration in this study; therefore, further analysis is required to determine which drugs are more hazardous.

## 5. Conclusions

In conclusion, this population-based cohort study revealed that opioid use was associated with an increased incidence of MACEs, such as AMI, HF, and stroke. Moreover, opioid use was associated with an increased risk of cardiovascular mortality. These associations were evident in both short- and long-term opioid users. The findings indicate that the use of an opioid prescription may elevate the risk of MACEs in adults, highlighting the need to weigh the benefits and risks associated with their prescription. Our findings indicate that preventing the abuse or misuse of opioids is crucial for reducing the incidence of MACEs and associated mortality. However, the study results should be interpreted with caution, as the high incidence of MACEs might not be due only to opioid exposure, and other factors may have influenced the outcome. Future studies are needed to confirm these findings.

## Figures and Tables

**Table 1:** The clinicopathological characteristics of the study population before and after PS matching.

Variable		Before PS Matching		ASD	*p*-Value	After PS Matching		ASD	*p*-Value
		Opioid User *n* = 1,882,945	Non-User *n* = 2,296,185			Opioid User *n* = 905,860	Non-User *n* = 905,860		
Age—year		54.6 (16.0)	54.8 (16.2)	0.076	<0.001	55.9 (15.8)	55.3 (15.9)	0.029	<0.001
Sex—male		871,918 (46.3)	1,079,594 (47.0)	0.006	<0.001	410,521 (45.3)	352,745 (38.9)	0.108	<0.001
Household income level					<0.001				<0.001
	Medical aid program group	82,859 (4.4)	70,528 (3.1)			43,333 (4.8)	29,346 (3.2)		
	Q1 in quartile (lowest)	349,393 (18.6)	417,107 (18.2)	0.017		173,037 (19.1)	166,218 (18.3)	0.043	
	Q2 in quartile	384,330 (20.4)	449,852 (19.6)	0.002		185,500 (20.5)	174,815 (19.3)	0.027	
	Q3 in quartile	463,848 (24.6)	549,213 (23.9)	0.054		220,482 (24.3)	222,323 (24.5)	0.091	
	Q4 in quartile (highest)	571,655 (30.4)	768,834 (33.5)	0.066		264,965 (29.3)	297,239 (32.8)	0.108	
	Unknown	30,860 (1.6)	40,651 (1.8)	<0.001		18,543 (2.0)	15,925 (1.8)	0.009	
Residence					<0.001				<0.001
	Urban area	778,181 (41.3)	1,049,224 (45.7)			373,587 (41.2)	415,449 (45.9)		
	Rural area	1,104,764 (58.7)	1,246,961 (54.3)	0.004		532,273 (58.8)	490,417 (54.1)	0.133	
Underlying disability					<0.001				<0.001
	Mild to moderate	114,891 (6.1)	99,554 (4.3)	0.197		63,705 (7.0)	42,077 (4.6)	0.108	
	Severe	40,162 (2.1)	52,994 (2.3)	0.135		22,371 (2.5)	16,077 (1.8)	0.049	
Underlying comorbidity									
	Congestive heart failure	48 (0.0)	37 (0.0)	<0.001	<0.001	25 (0.0)	19 (0.0)	0.001	0.366
	Cardiac arrhythmias	53,804 (2.9)	49,225 (2.1)	0.039	<0.001	30,435 (3.4)	23,953 (2.6)	0.062	<0.001
	Valvular disease	5225 (0.3)	5925 (0.3)	0.004	<0.001	2873 (0.3)	2744 (0.3)	0.011	0.085
	Pulmonary circulation disorders	3012 (0.2)	2597 (0.1)	0.004	<0.001	1607 (0.2)	1175 (0.1)	0.015	<0.001
	Peripheral vascular disorders	258,278 (13.7)	175,580 (7.6)	0.270	<0.001	141,217 (15.6)	87,901 (9.7)	0.169	<0.001
	Hypertension—uncomplicated	578,421 (30.7)	596,215 (26.0)	0.097	<0.001	318,684 (35.2)	264,877 (29.2)	0.136	<0.001
	Hypertension—complicated	33,356 (1.8)	35,560 (1.5)	0.013	<0.001	18,854 (2.1)	15,712 (1.7)	0.027	<0.001
	Paralysis	5691 (0.3)	6597 (0.3)	0.001	<0.001	2723 (0.3)	1600 (0.2)	0.024	<0.001
	Other neurological disorders	71,814 (3.8)	61,355 (2.7)	0.057	<0.001	38,012 (4.2)	25,158 (2.8)	0.068	<0.001
	Chronic pulmonary disease	597,851 (31.8)	476,427 (20.7)	0.235	<0.001	317,714 (35.1)	298,210 (32.9)	0.077	<0.001
	Diabetes—uncomplicated	307,581 (16.3)	290,710 (12.7)	0.349	<0.001	173,258 (19.1)	133,616 (14.8)	0.123	<0.001
	Diabetes—complicated	150,712 (8.0)	141,105 (6.1)	0.253	<0.001	80,608 (8.9)	62,674 (6.9)	0.075	<0.001
	Hypothyroidism	94,623 (5.0)	89,752 (3.9)	0.057	<0.001	53,598 (5.9)	46,476 (5.1)	0.045	<0.001
	Renal failure	21,403 (1.1)	24,979 (1.1)	0.002	<0.001	11,894 (1.3)	9766 (1.1)	0.024	<0.001
	Liver disease	443,345 (23.5)	382,313 (16.6)	0.162	<0.001	253,233 (28.0)	185,168 (20.4)	0.195	<0.001
	PUD excluding bleeding	395,484 (21.0)	274,756 (12.0)	0.204	<0.001	222,997 (24.6)	149,217 (16.5)	0.188	<0.001
	AIDS/HIV	1071 (0.1)	1088 (0.0)	0.006	<0.001	586 (0.1)	481 (0.1)	0.005	<0.001
	Lymphoma	2804 (0.1)	2968 (0.1)	0.007	<0.001	1488 (0.2)	1431 (0.2)	0.010	0.001
	Metastatic cancer	14,323 (0.8)	14,590 (0.6)	0.020	<0.001	8253 (0.9)	6228 (0.7)	0.021	0.291
	Solid tumor without metastasis	114,615 (6.1)	128,061 (5.6)	0.023	<0.001	64,746 (7.1)	57,899 (6.4)	0.034	<0.001
	RA/collagen vascular diseases	135,427 (7.2)	67,320 (2.9)	0.168	<0.001	67,667 (7.5)	35,842 (4.0)	0.139	<0.001
	Coagulopathy	16,185 (0.9)	15,001 (0.7)	0.021	<0.001	9167 (1.0)	6836 (0.8)	0.027	<0.001
	Obesity	2917 (0.2)	1944 (0.1)	0.019	<0.001	1748 (0.2)	1042 (0.1)	0.027	<0.001
	Weight loss	12,671 (0.7)	12,002 (0.5)	0.025	<0.001	7100 (0.8)	5204 (0.6)	0.022	<0.001
	Fluid and electrolyte disorders	93,424 (5.0)	73,234 (3.2)	0.070	<0.001	52,296 (5.8)	38,198 (4.2)	0.097	<0.001
	Blood loss anemia	4156 (0.2)	3776 (0.2)	0.007	<0.001	2317 (0.3)	1811 (0.2)	<0.001	<0.001
	Deficiency anemia	100,002 (5.3)	90,066 (3.9)	0.057	<0.001	56,345 (6.2)	43,263 (4.8)	0.072	<0.001
	Alcohol abuse	37,676 (2.0)	32,616 (1.4)	0.040	<0.001	21,323 (2.4)	12,919 (1.4)	0.068	<0.001
	Drug abuse	857 (0.0)	593 (0.0)	0.014	<0.001	425 (0.0)	273 (0.0)	0.013	<0.001
	Psychoses	15,564 (0.8)	23,169 (1.0)	0.094	<0.001	8851 (1.0)	6809 (0.8)	0.034	<0.001
	Depression	217,050 (11.5)	143,764 (6.3)	0.151	<0.001	115,744 (12.8)	72,177 (8.0)	0.151	<0.001
Prescription of other analgesics									
	Paracetamol	1,869,282 (99.3)	942,072 (41.0)	6.167	<0.001	892,877 (98.6)	892,885 (98.6)	<0.001	0.990
	NSAIDs	643,414 (34.2)	524,596 (22.8)	0.238	<0.001	336,891 (37.2)	301,379 (33.3)	0.109	<0.001
	Gabapentin or pregabalin	167,446 (8.9)	47,620 (2.1)	0.242	<0.001	46,068 (5.1)	26,555 (2.9)	0.095	<0.001

PS—propensity score; ASD—absolute value of standardized mean difference; PUD—peptic ulcer disease; AIDS—acquired immunodeficiency syndrome; HIV—human immunodeficiency virus; RA—rheumatoid arthritis; NSAIDs—nonsteroidal anti-inflammatory drugs.

**Table 2:** Analysis before and after PS matching.

Variable	Event (*n*, %)	HR (95% CI)	*p*-Value
Before PS matching			
MACE in 2017–2021			
Non-user	339,824/2,296,185 (14.8)	1	
Opioid user	349,860/1,882,945 (18.6)	1.25 (1.25, 1.26)	<0.001
AMI			
Non-user	62,197/2,296,185 (2.7)	1	
Opioid user	68,303/1,882,945 (3.6)	1.33 (1.32, 1.35)	<0.001
HF			
Non-user	228,624/2,296,185 (10.0)	1	
Opioid user	240,851/1,882,945 (12.8)	1.28 (1.27, 1.29)	<0.001
Stroke			
Non-user	126,604/2,296,185 (5.5)	1	
Opioid user	125,534/1,882,945 (6.7)	1.21 (1.20, 1.21)	<0.001
CV mortality			
Non-user	18,689/2,296,185 (0.8)	1	
Opioid user	13,279/1,882,945 (0.7)	0.87(0.85, 0.88)	<0.001
After PS matching			
MACE in 2017–2021			
Non-user	148,565/905,866 (16.4)	1	
Opioid user	181,448/905,866 (20.0)	1.24 (1.23, 1.24)	<0.001
AMI			
Non-user	27,214/905,866 (3.0)	1	
Opioid user	35,415/905,866 (3.9)	1.32 (1.30, 1.34)	<0.001
HF			
Non-user	100,868/905,866 (11.1)	1	
Opioid user	125,076/905,866 (13.8)	1.25 (1.24, 1.27)	<0.001
Stroke			
Non-user	53,486/905,866 (5.9)	1	
Opioid user	64,906/905,866 (7.2)	1.23 (1.21, 1.24)	<0.001
CV mortality			
Non-user	5320/905,860 (0.6)	1	
Opioid user	6875/905,860 (0.8)	1.30 (1.26, 1.35)	<0.001

PS—propensity score; HR—hazard ratio; CI—confidence interval; MACE—major adverse cardiovascular event; AMI—acute myocardial infarction; HF—heart failure; CV—cardiovascular.

**Table 3:** Multivariable Cox regression model for MACEs or cardiovascular mortality in 2017–2021.

Variable	HR (95% CI)	*p*-Value
MACE		
Multivariable model 1		
Non-users	1	
Opioid user	1.12 (1.10, 1.12)	<0.001
Multivariable model 2		
Non-users	1	
Short-term opioid user	1.10 (1.09, 1.10)	<0.001
Long-term opioid user	1.18 (1.17, 1.19)	<0.001
AMI		
Multivariable model 3		
Non-users	1	
Opioid user	1.16 (1.15, 1.18)	<0.001
Multivariable model 4		
Non-users	1	
Short-term opioid user	1.14 (1.12, 1.15)	<0.001
Long-term opioid user	1.23 (1.21, 1.25)	<0.001
HF		
Multivariable model 5		
Non-users	1	
Opioid user	1.12 (1.11, 1.13)	<0.001
Multivariable model 6		
Non-users	1	
Short-term opioid user	1.09 (1.08, 1.10)	<0.001
Long-term opioid user	1.18 (1.17, 1.19)	<0.001
Stroke		
Multivariable model 7		
Non-users	1	
Opioid user	1.09 (1.08, 1.10)	<0.001
Multivariable model 8		
Non-users	1	
Short-term opioid user	1.07 (1.05, 1.08)	<0.001
Long-term opioid user	1.13 (1.12, 1.15)	<0.001
CV mortality		
Multivariable model 9		
Non-users	1	
Opioid user	1.19 (1.15, 1.22)	<0.001
Multivariable model 10		
Non-users	1	
Short-term opioid user	1.11 (1.07, 1.14)	<0.001
Long-term opioid user	1.32 (1.27, 1.37)	<0.001

All covariates in Table 1 were used for multivariable adjustment in 10 models. Models 1 and 2 endpoint—total MACEs; models 3 and 4 endpoint—AMI; models 5 and 6 endpoint—HF; models 7 and 8 endpoint—stroke; models 9 and 10 endpoint—CV mortality. MACE—major adverse cardiovascular event; HR—hazard ratio; CI—confidence interval; AMI—acute myocardial infarction; HF—heart failure; CV—cardiovascular.

## Data Availability

Data will be available upon request to the corresponding author.

## Supplementary Materials

The following supporting information can be downloaded at https://www.mdpi.com/article/10.3390/jcm14041205/s1, Table S1: All HRs with 95% Cis of other covariates in multivariable model 1. Table S2: All HRs with 95% Cis of other covariates in multivariable model 3.

## Abbreviations

MACE	major adverse cardiovascular event
HR	hazard ratio
CI	confidence interval
AMI	acute myocardial infarction
HF	heart failure
NHIS	National Health Insurance Agency
ICD-10	International Classification of Diseases, 10th Revision
SD	standard deviation
PS	propensity score
ASD	absolute value of the standardized mean difference
