# Supplementary material for: Association of Opioid Prescription with Major Adverse Cardiovascular Events: Nationwide Cohort Study
Source: J Clin Med. 2025 Feb 12;14(4):1205. doi: 10.3390/jcm14041205 (PMC11855939; doi:10.3390/jcm14041205)
Supplement: Supplementary file 1 [file jcm-14-01205-s001.zip › Table S1.pdf]

Table S1. All HRs with 95% Cis of other covariates in multivariable models 1

Variable	HR (95% CI)	*p*-value
Age, year	1.05 (1.05, 1.05)	<0.001
Sex, male	1.13 (1.13, 1.14)	<0.001
Household income level		
Medical aid program group	1.34 (1.32, 1.36)	<0.001
Q1 in quartile (lowest)	1	
Q2 in quartile	0.99 (0.98, 0.99)	0.021
Q3 in quartile	0.97 (0.96, 0.98)	<0.001
Q4 in quartile (highest)	0.93 (0.92, 0.94)	<0.001
Unknown	1.02 (1.00, 1.05)	0.058
Residence		
Urban area	1	
Rural area	1.12 (1.12, 1.13)	<0.001
Underlying disability		
Mild to moderate	1.26 (1.25, 1.28)	<0.001
Severe	1.49 (1.47, 1.52)	<0.001
Underlying comorbidity		
Congestive heart failure	1.39 (0.86, 2.25)	0.182
Cardiac arrhythmias	1.71 (1.69, 1.73)	<0001
Valvular disease	2.52 (2.2, 2.63)	<0.001
Pulmonary circulation disorders	1.38 (1.30, 1.46)	<0.001
Peripheral vascular disorders	1.18 (1.17, 1.19)	<0.001
Hypertension, uncomplicated	1.42 (1.41, 1.43)	<0.001
Hypertension, complicated	1.50 (1.47, 1.52)	<0.001
Paralysis	1.24 (1.19, 1.29)	<0.001

Other neurological disorders	1.28 (1.26, 1.29)	<0.001
Chronic pulmonary disease	1.20 (1.19, 1.20)	<0.001
Diabetes, uncomplicated	1.15 (1.14, 1.15)	<0.001
Diabetes, complicated	1.23 (1.22, 1.24)	<0.001
Hypothyroidism	1.07 (1.06, 1.08)	<0.001
Renal failure	1.43 (1.40, 1.46)	<0.001
Liver disease	1.10 (1.09, 1.11)	<0.001
Peptic ulcer disease, excluding bleeding	1.14 (1.13, 1.15)	<0.001
AIDS/HIV	1.18 (1.05, 1.33)	0.005
Lymphoma	1.13 (1.07, 1.21)	<0.001
Metastatic cancer	0.90 (0.87, 1.11)	0.125
Solid tumor without metastasis	1.06 (1.05, 1.07)	<0.001
Rheumatoid arthritis/collagen vascular diseases	1.14 (1.13, 1.15)	<0.001
Coagulopathy	1.23 (1.22, 1.24)	<0.001
Obesity	1.18 (1.09, 1.28)	<0.001
Weight loss	1.06 (1.03, 1.10)	<0.001
Fluid and electrolyte disorders	1.20 (1.18, 1.21)	<0.001
Blood loss anemia	1.14 (1.08, 1.21)	<0.001
Deficiency anemia	1.07 (1.06, 1.08)	<0.001
Alcohol abuse	1.27 (1.24, 1.29)	<0.001
Drug abuse	1.32 (1.17, 1.50)	<0.001
Psychoses	1.04 (1.02, 1.07)	0.002
Depression	1.37 (1.36, 1.38)	<0.001
Prescription of other analgesics		
Paracetamol	1.09 (1.09, 1.10)	<0.001
NSAIDs	1.05 (1.05, 1.06)	<0.001
Gabapentin or pregabalin	1.15 (1.14, 1.16)	<0.001

HR, hazard ratio; CI, confidence interval; AIDS, acquired immunodeficiency syndrome; HIV, human immunodeficiency virus; NSAIDs, nonsteroidal anti-inflammatory drugs
