# Supplementary material for: Association of Opioid Prescription with Major Adverse Cardiovascular Events: Nationwide Cohort Study
Source: J Clin Med. 2025 Feb 12;14(4):1205. doi: 10.3390/jcm14041205 (PMC11855939; doi:10.3390/jcm14041205)
Supplement: Supplementary file 1 [file jcm-14-01205-s001.zip › Table S2.pdf]

Table S2. All HRs with 95% Cis of other covariates in multivariable models 3

Variable	HR (95% CI)	*p*-value
Age, year	1.13 (1.13, 1.13)	<0.001
Sex, male	1.56 (1.53, 1.60)	<0.001
Household income level		
Medical aid program group	1.39 (1.33, 1.45)	<0.001
Q1 in quartile (lowest)	1	
Q2 in quartile	1.00 (0.96, 1.03)	0.793
Q3 in quartile	0.89 (0.85, 0.92)	<0.001
Q4 in quartile (highest)	0.78 (0.76, 0.81)	<0.001
Unknown	0.83 (0.75, 0.81)	<0.001
Residence		
Urban area	1	
Rural area	1.04 (1.02, 1.07)	<0.001
Underlying disability		
Mild to moderate	1.25 (1.21, 1.29)	<0.001
Severe	2.11 (2.03, 2.20)	<0.001
Underlying comorbidity		
Congestive heart failure	1.86 (0.60, 5.77)	0.282
Cardiac arrhythmias	1.40 (1.34, 1.47)	<0.001
Valvular disease	2.24 (2.05, 2.45)	<0.001
Pulmonary circulation disorders	1.58 (1.36, 1.84)	<0.001
Peripheral vascular disorders	0.97 (0.95, 1.00)	0.045
Hypertension, uncomplicated	1.29 (1.26, 1.32)	<0.001
Hypertension, complicated	1.00 (0.94, 1.05)	0.847
Paralysis	3.10 (2.88, 3.33)	<0.001

Other neurological disorders	1.31 (1.26, 1.36)	<0.001
Chronic pulmonary disease	0.98 (0.96, 1.01)	0.120
Diabetes, uncomplicated	1.05 (1.02, 1.08)	0.001
Diabetes, complicated	1.21 (1.17, 1.25)	<0.001
Hypothyroidism	0.82 (0.78, 0.87)	<0.001
Renal failure	1.56 (1.48, 1.65)	<0.001
Liver disease	0.85 (0.82, 0.87)	<0.001
Peptic ulcer disease, excluding bleeding	0.87 (0.84, 0.89)	<0.001
AIDS/HIV	0.76 (0.40. 1.47)	0.418
Lymphoma	0.87 (0.66, 1.14)	0.300
Metastatic cancer	0.93 (0.80, 1.08)	0.358
Solid tumor without metastasis	0.75 (0.72, 1.05)	0.085
Rheumatoid arthritis/collagen vascular diseases	0.98 (0.93, 1.03)	0.347
Coagulopathy	1.09 (0.99, 1.21)	0.081
Obesity	0.66 (0.34, 1.26)	0.208
Weight loss	1.14 (1.04, 1.26)	0.005
Fluid and electrolyte disorders	1.32 (1.26, 1.37)	<0.001
Blood loss anemia	1.20 (0.99, 1.46)	0.059
Deficiency anemia	1.20 (1.16, 1.25)	<0.001
Alcohol abuse	1.59 (1.48, 1.71)	<0.001
Drug abuse	1.30 (0.89, 1.92)	0.178
Psychoses	1.69 (1.58, 1.80)	<0.001
Depression	1.18 (1.15, 1.22)	<0.001
Prescription of other analgesics		
Paracetamol	0.64 (0.62, 0.66)	<0.001
NSAIDs	0.76 (0.74, 0.79)	<0.001
Gabapentin or pregabalin	0.94 (0.91, 0.98)	0.006

HR, hazard ratio; CI, confidence interval; AIDS, acquired immunodeficiency syndrome; HIV, human immunodeficiency virus; NSAIDs, nonsteroidal anti-inflammatory drugs
